# Whole-Genome Sequence of the Soil Bacterium *Micrococcus* sp. KBS0714

**DOI:** 10.1128/genomeA.00697-17

**Published:** 2017-08-10

**Authors:** V. Kuo, W. R. Shoemaker, M. E. Muscarella, J. T. Lennon

**Affiliations:** a Department of Biology, Indiana University, Bloomington, Indiana, USA; b Department of Plant Biology, University of Illinois, Urbana-Champaign, Illinois, USA

## Abstract

We present here a draft genome assembly of *Micrococcus* sp. KBS0714, which was isolated from agricultural soil. The genome provides insight into the strategies that *Micrococcus* spp. use to contend with environmental stressors such as desiccation and starvation in environmental and host-associated ecosystems.

## GENOME ANNOUNCEMENT

Microorganisms have evolved traits that allow them to cope with a wide range of environmental conditions. For example, chemoorganotrophic representatives of the genus *Micrococcus* (class *Actinobacteria*, family *Micrococcaceae*) are commonly found in temperate soil (1), water (2), mammalian skin (3), Antarctic ice (4), and desert soil (5). Survival and reproduction in some of these habitats has been linked to the ability of *Micrococcus* spp. to form biofilms or enter dormant stages in response to conditions such as desiccation and starvation (6–8). Genome sequencing may illuminate additional traits that allow *Micrococcus* spp. to persist when challenged with environmental stressors. However, outside of isolates from contaminated ecosystems (1, 9–12), very few *Micrococcus* genomes have been sequenced from soil. Here, we present the draft genome of *Micrococcus* sp. KBS0714, isolated from never-tilled agricultural soil at the Kellogg Biological Station Long-Term Ecological Research site (6, 13).

*Micrococcus* sp. KBS0714 genomic DNA was prepared with the Illumina TruSeq DNA sample prep kit using an insert size of 250 bp for sequencing on an Illumina HiSeq 2500 with 100-bp paired-end reads (Illumina, San Diego, CA, USA). Raw sequences were processed by removing the TruSeq adaptors and the first 10 bp using Cutadapt version 1.9 (14), interleaving the paired reads using khmer version 2.0 (15), and quality-filtering with an average Phred score of 30 using the FASTX-Toolkit version 0.0.13 (Hannon Lab, http://hannonlab.cshl.edu/fastx_toolkit). The coverage was normalized to 25 based on a *k*-mer size of 25 bp using khmer, resulting in a total of 1,614,974 unmapped paired-end reads. The genome was assembled using Velvet version 1.2.10 (16) with the following parameters: a *k*-mer size of 55, expected coverage of 18×, and a coverage cutoff of 2.29. Contigs longer than 200 bp were annotated using Prokka version 1.12 (https://github.com/tseemann/prokka) (17). Finally, we used MAPLE version 2.3.0 with bidirectional best-hit matches (http://www.genome.jp/tools/maple) (18) to predict metabolic and physiological functions.

The draft assembly of *Micrococcus* sp. KBS0714 comprises 2,489,009 bp. It consists of 63 contigs with an *N*_50_ of 122,407 and a G+C content of 69%. Based on our gene annotation, the genome contains 2,210 protein-coding sequences, 3 rRNAs, 52 tRNAs, 2,267 genes, and 2 transfer-messenger RNAs (tmRNAs).

The KBS0714 genome highlights traits that may be used by *Micrococcus* spp. in soil. For example, we found genes involved with general stress response (*relA*) (19, 20) and biofilm formation (*brpA*) (21) in both the genome of KBS0714 and the closely related *M. luteus* NCTC 2662 (99% 16S rRNA sequence similarity, NCBI CP001628). Additionally, we detected pathways for desiccation and starvation resistance, such as genes for osmotic stress response (*mtrB-mtrA*, *rpoE*) (22, 23), phosphate starvation response (*senX3*-*regX3*), and membrane lipid fluidity regulation (*desK*-*desR*) (24, 25). In summary, the KBS0714 genome has features that may provide a selective advantage under stressful conditions in soils and other environments.

### Accession number(s).

This draft genome assembly has been deposited in DDBJ/EMBL/GenBank under the accession number MVDF00000000. The version described here is the second version, MVDF02000000. The code used for assembly and annotation is available online at https://github.com/LennonLab/Micrococcus.
